# Range Management Affects Native Ungulate Populations in Península Valdés, a World Natural Heritage

**DOI:** 10.1371/journal.pone.0055655

**Published:** 2013-02-04

**Authors:** Marcela J. Nabte, Andrea I. Marino, María Victoria Rodríguez, Adrián Monjeau, Sergio L. Saba

**Affiliations:** 1 Centro Nacional Patagónico, Puerto Madryn, Chubut, Argentina; 2 Facultad de Ciencias Naturales, Universidad Nacional de la Patagonia San Juan Bosco, Puerto Madryn, Chubut, Argentina; 3 Consejo Nacional de Investigaciones Científicas y Técnicas, Buenos Aires, Argentina; 4 Programa de Ecofilosofía, Departamento de Filosofía, Fundación Bariloche, Bariloche, Río Negro, Argentina; University of Alberta, Canada

## Abstract

Sheep rearing is the main productive activity in Patagonian rangelands, where guanacos are the only native ungulate. Ranchers perceive a decrease in range carrying capacity as guanaco numbers increase, therefore guanaco conservation within private lands becomes a considerable challenge. This issue is particularly evident in the World Natural Heritage Península Valdés (PV), where there is a need to harmonize livestock production and biodiversity conservation. While sheep rearing prevails as the primary land use in the area, some ecotourism initiatives have been implemented to complement livestock production. In order to study how land use affected guanaco distribution, we characterized PV's ranches in terms of land subdivision, primary productivity, stocking-rate and management type, and assess how these variables affected guanaco encounter rates. Smaller ranches were composed of smaller paddocks (mean size 4.8 km^2^), which showed higher values of the remote-sensing derived Enhance Vegetation Index (EVI) (mean 0.14) and held higher sheep densities (mean 108.0 sheep/km^2^), while larger management units (mean size 23.8 km^2^), showed lower EVI values (mean 0.12) and lower stocking-rates (mean 36.7 sheep/km^2^). This pattern suggests that primary productivity has been a decisive factor to determine the minimal paddock size set by ranchers in PV, apparently precluding excessive land-subdivision in less productive areas. Guanaco encounter rate, expressed as number of guanacos per travelled kilometre, was inversely related to EVI and stocking-rate. However, land subdivision was the better predictor of guanaco encounter-rate within only sheep ranches, finding more guanacos per kilometre as paddock size increased. In contrast, in ranches where ecotourism was implemented as a complementary activity, guanaco encounter-rates were greater, regardless of paddock size. Our results suggest that the implementation of an additional activity by which landowners derive benefits from wildlife has prompted a beneficial outcome for guanacos, presumably through a decrease in harassment intensity. Finally, we propose possible mechanisms by which land subdivision may affect guanaco distribution and potential alternatives for the inclusion of wildlife conservation in a context of extensive livestock production.

## Introduction

In Argentina, protected areas for wildlife conservation in the Patagonian steppe occupy only a small fraction (4.62%) [1]. Since most biodiversity in this eco-region occurs outside these reserves, in order to plan conservation efforts, it is essential to consider the conflicts between wildlife and productive activities (i.e. any activity that produces a valued good or service) [2]. Land use often implies negative consequences for wildlife and these are particularly evident when the removal of certain species is supposed to increase landholders' incomes or reduce production costs [3]. For example, in many pastoral systems wild herbivores are perceived by ranchers as detrimental species that threaten livestock production due mainly to food competition, disease transmission and fence damage [4]. This conflict is exacerbated in arid and semiarid rangelands where forage and water availability show high spatial/temporal variability [5] intensifying competition and consequently, wild herbivore persecution.

Previous studies have addressed stocking rates [6], land subdivision [7] and land use type [5] among main variables affecting wildlife in arid and semiarid environments. Due to direct and indirect competition, wild herbivores abundance is often inversely correlated with livestock density [8], [9]. In addition, inadequate livestock management involving greater stocking rates than carrying capacity may promote forage and habitat degradation due to overgrazing [10]. Land subdivision may decrease overall carrying capacity and habitat heterogeneity, and augment habitat fragmentation [7], [11]. Additionally, increased land subdivision often implies increased human disturbance [12], [13]. All these effects might negatively impact wildlife and ecosystem functioning [3]. Regarding management practices, distinct land-use types might affect wildlife in different ways. Strict reserves or profitable initiatives involving wildlife (i.e. ecotourism or environmentally-certified production) are expected to improve conservation outcomes [2]. In contrast, landholders relying on entirely productive activities that implicate some wildlife-production conflict often lack the right incentives to compensate for the costs derived from wildlife tolerant practices [14]. Therefore, when resources are limited and competition turns significant, those ranchers are expected to be prone to reduce conflictive wildlife numbers within their ranges. Understanding how these anthropic factors shape distribution patterns of wild species at a pertinent spatial scale is essential to plan conservation actions that involve key herbivores in arid and semiarid rangelands.

The extra-Andean Patagonia comprises *c.* 750 000 km^2^ of arid and semiarid lands where extensive sheep ranching is one of the main productive activities. Unsustainable management practices during the last century have led to land degradation due to sheep overgrazing across most of the region [10], [15]. Regarding wildlife, top predators and herbivores have been hunted to reduce direct and indirect losses, and to feed shepherd dogs [16]. Guanacos *(Lama guanicoe)* are the only native ungulate in this ecosystem and, in addition to puma *(Puma concolor)* and culpeo foxes (*Pseudalopex culpaeus*), are considered the most conflictive species [17]–[19]. Guanaco and sheep diets overlap significantly [20] and landowners perceive a decrease in range carrying capacity as guanaco abundance increases [21]. This perception is amplified during drought periods and in degraded areas, which also intensify guanaco-sheep competition for forage and water. As a result, guanacos are heavily hunted or pushed out from ranches. This statement is supported by studies across Patagonian rangelands which have shown that guanacos and sheep densities are inversely correlated and that guanacos are displaced to marginal habitats [8], [9].

Since local perception of the species is so negative, guanaco conservation within private lands is problematic, even when ranches are located inside protected areas where more tolerant attitudes to wildlife would be expected. This issue is particularly evident in Península Valdés (PV), where both production and conservation need to be considered. Although some ranches have recently implemented ecotourism initiatives, sheep production is still the prevalent economic activity in the area. PV poses an interesting case study because extensive sheep production is managed in a similar way across most ranches but management units show a considerable heterogeneity in size, primary productivity and stocking rates. The aim of this study was to address guanaco distribution within PV in relation to land use practices and primary productivity. In order to carry out our objective, we conducted a characterization of PV's ranches in terms of stocking rate, primary productivity, land subdivision (ranch and paddocks size) and management type (only sheep rearing or sheep rearing and ecotourism). We then assessed how these variables affected guanaco encounter rates. Our main expectations were that 1) due to interspecific competition, guanacos abundance would be inversely related to stocking rate; 2) guanacos are displaced to marginal habitats in the presence of sheep, therefore guanaco abundance would be inversely related to primary production; 3) because of increased anthropic disturbance and/or reduced carrying capacity coupled to land subdivision, guanacos would be less abundant in smaller management units; and 4) as extra incomes derived from wildlife watching would encourage more tolerant attitudes towards conflictive species, ranches that included ecotourism as a complementary activity to sheep production would hold more guanacos than only sheep ranches.

## Methods

### Study area

**Figure 1:**
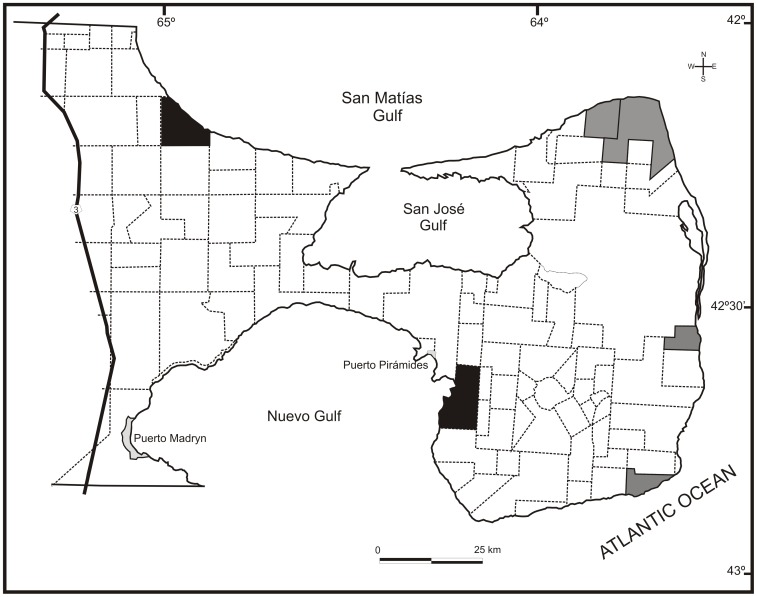
Península Valdés, Chubut, Argentina. Ranches' boundaries are indicated by dashed lines. Land use is indicated by color: sheep (white), sheep/ecotourism (grey) ranches, and wildlife reserves (black).

The study was conducted at PV, located in the northeastern province of Chubut. Its 400 000 ha are situated between 42° and 42° 45′ latitude S and 63° 35′ and 65° 17′W (Fig. 1). Average annual rainfall decreases towards the interior of the peninsula, ranging from 225 mm at the periphery to 200 mm in the central area [22]. The average annual temperature is 12.6°C [23]. In 1999, the protected area was declared a Natural Heritage site by UNESCO and according to the classification of conservation units of IUCN, it has been categorized as VI (Managed Resource Protected Area). Private ranches compose 98% of PV's land surface, 90 of them (94%) are exclusively managed as extensive sheep-productions, 4 (4%) have implemented mixed management (sheep rearing and ecotourism) and 2 have been converted into strict wildlife reserves. A typical sheep ranch is divided into a series of irregular paddocks of variable size (average size 13.3 km^2^, ranging from 1.3 to 40.4), delimited by fences one meter high to prevent sheep moving, though adult guanacos are able to jump over them. A single permanent water point is common in each paddock and the water availability is ensured by wind-driven pumping of underground water. At the time of this study, guanaco densities across PV ranged from a minimum of 1.10±0.53 guanacos/km^2^ within private ranches located at the PV Southern section to a maximum of 12.95±4.14 inside strict reserves [24], whereas average sheep density was 64.4±19.6 sheep/km^2^.

### Interview survey

In order to obtain information about management practices and land subdivision, a semi-structured oral interview survey was carried out throughout PV from August 2005 to March 2007 (n = 74). The identity of respondents was kept confidential, and interview records and notes were protected by the confidentiality agreement with the participants and laid in the Centro Nacional Patagónico. These surveys were conducted with the permission (Exp. N° 001339-OPT/05 Res. N° 052/05OPT; Disp. 021/05-DGCAP) of Dirección General de Conservación de Áreas Protegidas, Subsecretaría de Turismo y Áreas Protegidas and Dirección de Fauna y Flora de Chubut. In the absence of an ethical committee or IRB, ethical considerations are included in the permit issued by the government. These were discussed with the Advisory Council of the protected area before the permit was granted. No research was conducted outside our country of residence. Written consent was not obtained because it is not necessary, the collaboration of the participants is voluntary and consent to be interviewed is oral. No obligation or reward has encouraged participants to collaborate. The government, in granting the permit, has accepted the interview process, which is commonly accepted in the rural communities of Patagonia. Our experience indicates that the requirement of written consent may result in the refusal of some rural people to be interviewed and cause biases in the results. Ranchers were interviewed by the same person. At the beginning of each survey we drew a scheme of the ranch's paddocks, following the instructions of the rural settler. These schemes were based on a preliminary map previously derived from satellite Landsat TM and Google Earth images. Each interview provided information about sheep abundance per paddock and ranch management type (only sheep rearing/sheep rearing and ecotourism). Paddock size was then estimated from digitalized paddock schemes. Since continuous grazing is the usual management practice across PV, we expected stocking rate to reflect grazing pressure. Then, stoking rate per paddock was calculated dividing the number of sheep by the size of the paddock, and was expressed as number of sheep/km^2^. We collected data from 339 paddocks across 74 ranches, 77% of the 96 ranches that compose PV. Main ranch features are shown in Table 1. Our sample include the four (100%) ranches that have implemented mixed management and 70 (78%) of the ranches devoted exclusively to sheep production.

**Table 1:** Ranch features summary.

Management	Paddock size (km^2^)	Stocking rate (sheep/km^2^)	N° of dogs/ranch	Sample sizes	
				Paddocks	Ranches
Sheep ranching	13.7 (6.4)	45.5 (56.9)	7.7 (2.1)	307	70
Sheep ranching and ecotourism	11.3 (6.2)	53 (19.9)	3.5 (1.3)	32	4

Average values and standard deviations (SD).

### Vegetation Indexes

Enhanced Vegetation Index (EVI) derived from 250 m MODIS satellite images was used as an indicator of primary productivity [25]. These data are distributed by the Land Processes Distributed Active Archive Center (LP DAAC) (lpdaac.usgs.gov). Images corresponding to September 2007 were included in the GIS to account for the peak of primary productivity, which had been previously identified from monthly values across an annual phenological cycle. At this time of year, perennial grasses preferred as well as non-preferred by guanacos and sheep, show maximal vegetative growth rates. In general, shrubs also show high vegetative growth rates during this month. Therefore, given the high degree of synchronization of most functional types in their phenological cycle [26], EVI spring values are probably reflecting the productivity peak of all plants. Then, pixel values from each paddock were extracted and averaged. Thus the average EVI value at the peak of the greening season was considered as an indicator of relative primary productivity of each paddock. Paddocks near the coast that contained mixed pixels [25] as well as paddocks that did not hold sheep at the moment of the study were eliminated from the data set.

### Guanaco surveys

We conducted line-transect surveys to assess guanaco distribution during September 2006 in the western section of PV (west of Ameghino isthmus) and during October 2007 in the eastern section (east of Ameghino isthmus). We assumed that there was no significant movement of animals between both sections during the study period because the Ameghino isthmus acts as a natural bottleneck (Fig. 1) and later abundance estimations at local scale were consistent with estimations performed previous to the sampling period. We surveyed 107 paddocks located across 47 (50%) of the 94 ranches with production-oriented management. Surveys were conducted from an open pick-up vehicle with two observers standing in the back, using the distance sampling method [27]. For every guanaco group encountered we stopped the vehicle, recorded the number of animals, the perpendicular distance (measured using a laser rangefinder) from the transect line to the location where the group was standing at the time it was detected, and the observers location with a GPS. Survey trajectory as well as locations of the observed groups were included in an Geographic Information System (GIS) and overlaid with paddock maps, obtaining a record of guanaco encounter rate (guanaco per traveled kilometre) for each paddock surveyed.

### Statistical analysis

To assess the relationships among paddock features we fitted Linear Mixed Models. Firstly, we modeled paddock size as a function of EVI values. Secondly, we modeled sheep stocking rate as a function of EVI values and paddock size. All models included ranch identity as a random factor to account for the lack of independence between paddocks of the same ranch [28]. Response variables were log transform when necessary to meet model assumptions. We used *t* tests to assess the significance of the differences between factor levels or slopes of the fixed factors and variables, considering an *alpha* level of 0.05. When various models showed significant or nearly significant results, we used Akaike Information Criteria (AIC) to select the final model [28]. We selected the model with the lowest AIC and if the delta AIC<2, we selected the simplest model.

To address factors affecting guanaco distribution we fitted a set of Linear Mixed Models to the encounter-rate data. Encounter rate was expressed as number of guanacos observed per kilometre within each paddock surveyed. Raw data was log transformed after adding 1 to cope with zeros. Log transformation of raw data performed better in terms of residuals patterns than fitting a negative binomial distribution to the error term, which is usually suggested for this type of data [28]. Fixed factors considered were stocking rate, EVI, paddock size and type of management. As in previous models, ranch identity was included as a random factor to account for the lack of independence between paddocks of the same ranch. Stocking rate was expressed as sheep equivalent per km^2^. Model fitting was performed using the nlme package and the 2.9.2 version of R (The R Foundation for Statistical Computing, www.r-project.org, verified 26 June 2012) software.

## Results

### Ranch characterization

**Figure 2:**
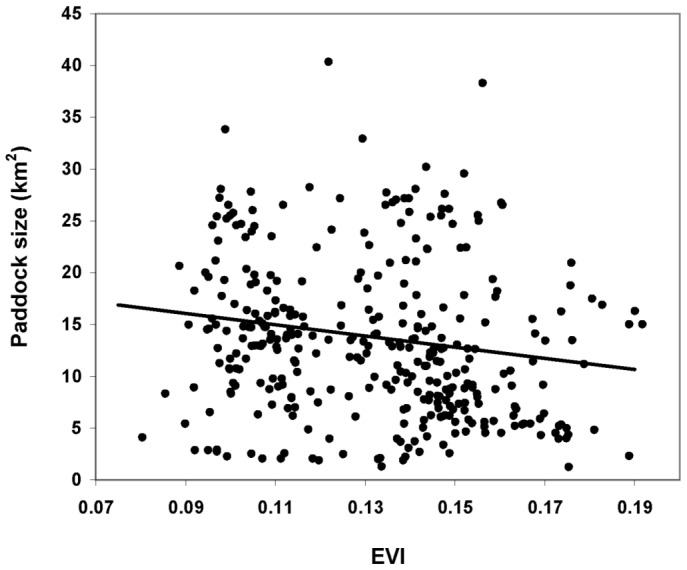
Predicted paddock size as a function of EVI values in Península Valdés.

The number of paddocks increased linearly with overall ranch area (Slope = 0.035 SE = 0.005 df = 78 p(t) = <.001), as well as the average paddock size (Slope = 0.072 SE = 0.013 df = 78 p(t) = <.001). We found a negative relationship between paddock size and EVI values (Slope = −53.96 SE = 20.19 df = 264 p(t) = 0.008; Fig. 2). The less productive paddocks, addressed by the intercept of the model, were on average about 20.92+−2.71 km^2^ whereas the 25% of the more productive ones (i.e. the fourth quartile of the data set increasingly ordered by EVI values) where on average about 10.97+−1.78 km^2^.

**Figure 3:**
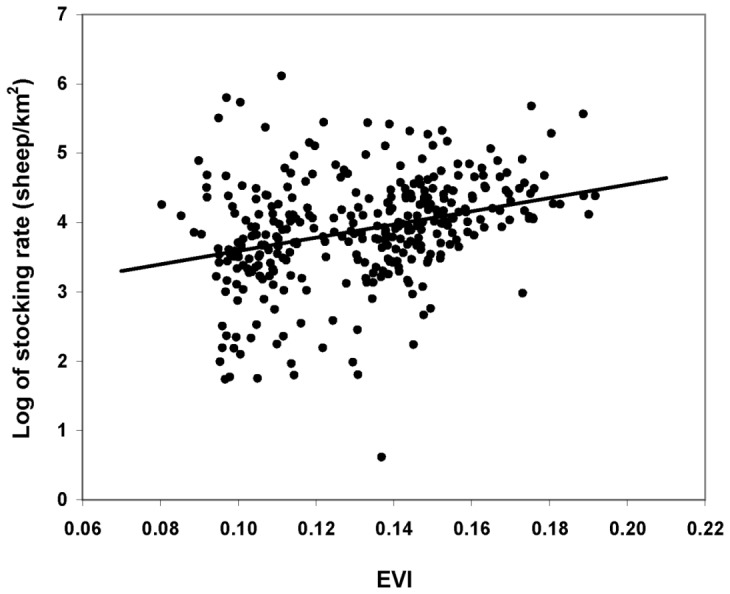
Predicted stocking rate as a function of EVI values in Península Valdés.

**Figure 4:**
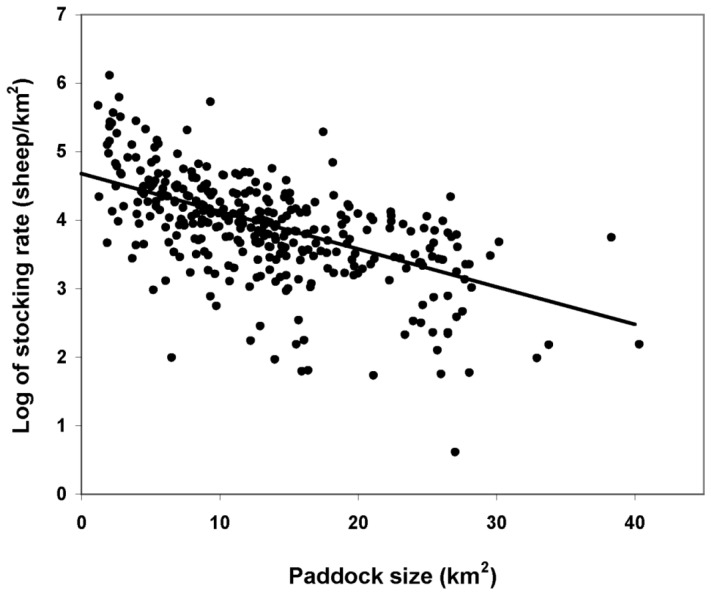
Predicted stocking rate as a function of paddock size in Península Valdés.

Even though multi-colinearity [28] between paddock size and EVI was expected given the relationship previously cited, the best model for stocking rate included both factors (AIC = 600.1) in contrast to individual models including only paddock size (AIC = 617.2) or including only EVI (AIC = 696.4). Correlation between parameters in the final model was relatively low (16%). Stocking rate was positively related to EVI values (Slope = 6.71 SE = 1.6 df = 263 p(t)<.001; Fig. 3) and inversely related to paddock size (Slope = −.0005 SE = .00004 df = 263 p(t)<.001; Fig. 4). There were no differences in stocking rate between ranches with traditional management and ranches with mixed management (Difference = 0.03 SE = 0.14 df = 262 p(t) = 0.797). Differences between paddocks of the same ranch accounted for 65% of the observed variation in stocking rates whereas differences between ranches accounted for the remaining 35%.

### Guanaco distribution

**Figure 5:**
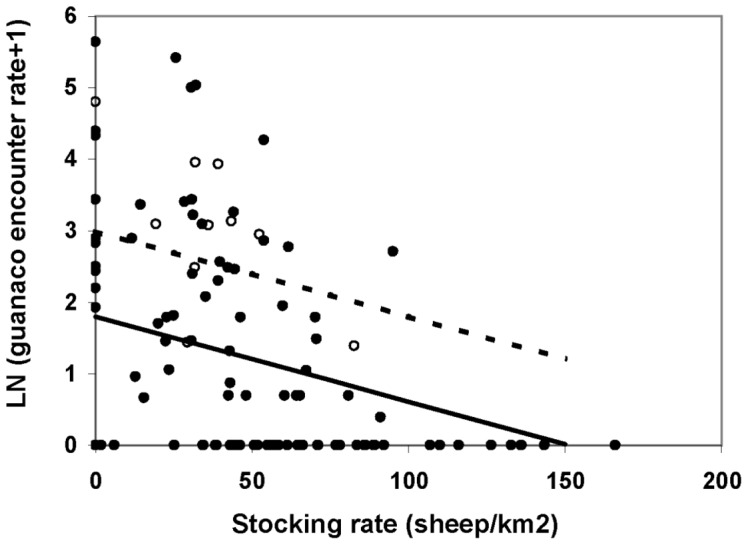
Predicted guanaco encounter rate as a function of sheep stocking rate in Península Valdés. Sheep ranches (solid line and black dots); Sheep/ecotourism ranches (dashed line and white dots).

**Figure 6:**
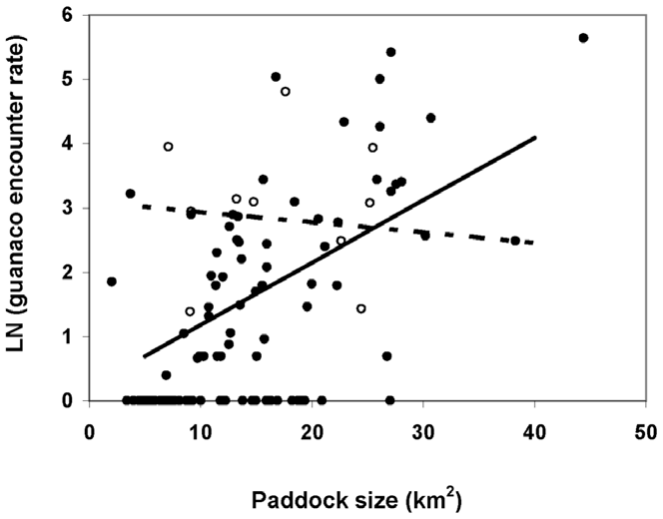
Predicted guanaco encounter rate as a function of paddock size in Península Valdés. Sheep ranches (solid line and black dots); sheep/ecotourism ranches (dashed line and white dots).

The final model selected for guanaco encounter rate included the effect of paddock size, management type and the interaction between them (AIC = 354.4). Guanaco encounter rate was inversely related to stocking rate (Fig. 5) but this effect was not significant if paddock size was considered in the same model (AIC = 353.2). Although stocking rate effect was significant if paddock size was not included, this model did not perform as well as the one including paddock size (AIC = 357.5). These results, which are consistent with the observed correlation between paddock size and stocking rate, designate paddock size as a better predictor of guanaco encounter rate than stocking rate. In ranches with mixed management, guanaco encounter rate was higher than in traditional ones (Difference = 3.21 SE = 1.09 df = 57 p(t) = 0.005) and was independent of paddock size (Slope = −0.0002 SE = .0006 df = 57 p(t) = 0.747; Fig. 6). In ranches with traditional management, guanaco encounter rate was low for the smallest paddocks but increased significantly with paddock size (Slope difference = .0012 SE = .0006 df = 57 p(t) = 0.049; Fig. 5). EVI had no effect on guanaco encounter rate (Slope difference = −8.99 SE = 5.47 df = 56 p(t) = 0.106). Regarding random terms, differences between ranches accounted for 37% of the observed variation in guanaco encounter rate whereas the remaining 63% was due to differences between paddocks of the same ranch.

## Discussion

We found noteworthy relationships among the variables considered in our characterization of the PV ranches. Primary productivity per unit area, stocking rate (sheep/km^2^) and land subdivision were positively correlated with each other across PV. The smaller ranches were composed of smaller paddocks, which showed relatively high primary productivity per unit area and held greater sheep densities. As expected, these ranches were concentrated in the southern portion of PV which is composed of highly productive grasslands, dominated by *Poa* and *Stipa* ssp., highly preferred by guanacos and sheep, and *Sporobolus rigens*, which is consumed intensively when the plants are young [20], [24]. In contrast, larger management units showed lower stocking rates and were located at central and northern sections of PV and the continental portion of the protected area, which are dominated by less productive shrublands [29]. The observed array suggests that primary productivity has been a decisive factor to determine land subdivision, presumably reflecting past intentions of maintaining management units large enough to keep sheep quantities at economically viable levels under the extensive grazing management adopted by Patagonian ranches. A similar pattern of land subdivision according to grazing management, primary productivity and/or production profitability has been previously described for several African and Asian grasslands [30], [31].

Regarding guanaco distribution, we found the expected correlates under our hypotheses. As predicted, guanaco encounter rate was inversely related to EVI and stocking rate. A similar pattern was found in previous studies at different scales [8], [9]. These results support the hypothesis that sheep, or the activities related to their production, have pushed guanacos to marginal habitats, as previously suggested by Baldi et al. [8]. Regarding the effect of land subdivision on guanaco distribution, we found different patterns between only sheep ranches and mix management ranches. Intriguingly, paddock size was a better predictor of guanaco encounter rate in only sheep ranches than EVI or stocking rate. Similar decreases in wildlife abundance as land subdivision increased were documented for other rangelands [7], [31] and were suspected to be the result of productivity loss due to reduced heterogeneity in smaller paddocks and/or limitations to animal displacements. In our study case, we propose three hypotheses to explain the higher guanaco abundance in larger paddocks: I- Previous studies conducted at PV and north-eastern Patagonia [32]–[35] indicated that as a consequence of a reduction in sheep grazing pressure, vegetation and perennial-grass cover were higher at sites far from water points (piosphere effect, [36]). As paddocks usually have only one water point, larger paddocks would contain a higher proportion of their surface less affected by sheep grazing. This heterogeneity in grazing pressure might offer wild herbivores a release from direct competition far from water points and could lead to a differential distribution of livestock and guanacos within paddocks. This was the case of wild herbivores and livestock in Kenya [12]. As this effect would be more pronounced in larger paddocks it could explain the positive correlation observed between paddock size and guanacos relative abundance. Therefore, in similar circumstances to those described in this study, heterogeneity in grazing pressure by livestock as a result of fixed distribution of water points would entail better conditions for wild herbivores. This hypothesis deserves attention because it opposes the notion that intensive rotational grazing is one of the most conservation-prone practices for these types of rangelands [11]. In this sense, numerous studies across the world provide evidence that spatial heterogeneity resulting from domestic grazing favours wildlife [37], [38]; II- On the other hand, paddocks of different sizes may differ in plant communities and shrub/grass ratio, as suggested by their EVI values and location. Diet overlap between guanacos and sheep might be higher in sites with high grass cover than in sites dominated by woody plants, where guanacos can switch to less palatable shrubs [8], [20]. Therefore, guanacos might be able to cope with direct competition easier in larger shrubby paddocks; III- Finally, larger paddocks may impose a constraint to harassment intensity (i.e. less dogs, hunters, roads and fences per surface unit) which might make it more difficult for locals to displace guanacos. This hypothesis is supported by the fact that in ranches where ecotourism is a complementary activity to wool production, guanaco encounter rate is higher than in only sheep ranches, and independent of paddock size. This last result suggests that guanacos in the smaller paddocks of the former would not be harassed with the same intensity than in only sheep paddocks of the same size. Ranchers' testimony is consistent with this idea (Nabte, unpublished data). In Kenya, de Leeuw et al., have suggested that farmers' activity disturbed wildlife [12]. Indeed, negative outcomes due to increased anthropic disturbance were documented for ostriches (*Struthio camelus*) and other birds [39], [40]. However, we failed to find a study assessing anthropic-disturbance effects in relation to land subdivision. Our three hypotheses are not mutually exclusive and probably these processes interact with each other to affect guanaco abundance inside PV ranches. Future studies will help to test these ideas and assess their relative contribution to understand the processes shaping guanaco distribution patterns within Patagonian rangelands.

### Conservation implications

Even though our results suggest that primary productivity has been a limiting factor to determine land subdivision at PV, excessive reduction of management units while maintaining extensive grazing systems might still have decreased the chances to reach economic profitability and/or to cope with environmental and market instability, threatening the already compromised sustainability of sheep production across the region. In 2009, the Ministerio de la Producción de la Provincia de Chubut (provincial ministry responsible for agriculture and livestock) recognized that in order to reach a minimum competitive level, a Patagonian ranch should hold between 6000–8000 sheep [41]. According to this criterion, most of the PV ranches seemed to be below a cost-effective level at the moment of this study, presumably due to a lack of the combination of size and productivity required to support the minimum profitable stock. Within this setting, finding productive alternatives to the current extensive practices, oriented to achieve ecological and economic sustainability becomes a priority. Even though the number of ranches with mixed management in PV is still low, we found that they hold significantly more guanacos than only sheep ranches. This difference suggests that the implementation of an extra activity such as ecotourism, by which landowners derive benefits from wildlife, has prompted a beneficial outcome for guanacos. The development of strategies that include wildlife use, mainly for recreational activities, for instance photographic safaris, in ranches with livestock production has been reported as an efficient approach to wildlife conservation while improving landowners' incomes in other regions [42], [43]. However, it would be improbable that all PV ranches could implement ecotourism. Other policies oriented to balance the costs and benefits derived from conservation efforts have shown to operate as efficient incentives, such as environmental certification [44], stewardship payments, tax concessions or other forms of sustainable use [45]. Proper incentives might not only increase the local's tolerance towards conflictive species improving native biodiversity but might complement traditional productive activities improving local economy [2], [3]. Among the former, environmental certification is a promising alternative for Patagonian wool producers although key species, such as dominant wild herbivores, still need to be included in certification standards in order to accomplish an ecosystemic approach to biodiversity conservation across extra-Andean Patagonia.
